# Microhand Platform Equipped with Plate-Shaped End-Effectors Enables Precise Probing of Intracellular Structure Contribution to Whole-Cell Mechanical Properties

**DOI:** 10.3390/mi16111272

**Published:** 2025-11-12

**Authors:** Masahiro Kawakami, Masaru Kojima, Toshihiko Ogura, Atsushi Kubo, Tatsuo Arai, Shinji Sakai

**Affiliations:** 1 Department of Materials Engineering Science, Division of Chemical Engineering, Graduate School of Engineering Science, The University of Osaka, 1-3 Machikaneyama-cho, Toyonaka 560-8531, Osaka, Japan; 2 Center for Cancer Immunotherapy and Immunobiology, Kyoto University, Yoshida-Konoe-cho, Sakyo-ku 606-8501, Kyoto, Japan; ogura.toshihiko.4n@kyoto-u.ac.jp; 3 Graduate School of Pharmaceutical Sciences, The University of Osaka, 1-6 Yamadaoka, Suita 565-0871, Osaka, Japan; 4 The Center for Neuroscience and Biomedical Engineering, The University of Electro-Communications, 1-5-1, Chofugaoka, Chofu 182-8585, Tokyo, Japan

**Keywords:** cell mechanics, micro-manipulation, HGPS

## Abstract

Cellular mechanical properties are critical indicators of cellular state and promising disease biomarkers. This study introduces a novel microhand system, featuring chopstick-like plate-shaped end-effectors, designed for stable and high-precision single-cell mechanical characterization. First, we automated the force sensor calibration to overcome the inefficiency and unreliability of conventional manual methods. To validate the system’s sensitivity, we precisely quantified the mechanical contributions of subcellular components, such as the actin cytoskeleton and chromatin, by measuring stiffness reductions after treatment with Cytochalasin D and Trichostatin A, respectively. Notably, when applied to a cellular model of Hutchinson–Gilford progeria syndrome, we successfully captured disease-induced mechanical alterations. A distinct population of high-stiffness cells was detected in progerin-overexpressing cells, a feature not observed in the control groups. Furthermore, by controlling the indentation speed and depth, the mechanical properties of the cytoplasm and nucleus could be distinctly evaluated. These results demonstrate that our microhand system is a highly sensitive and robust platform, capable of detecting subtle, disease-related changes and elucidating the contributions of specific subcellular structures to cell mechanics.

## 1. Introduction

Cell mechanical properties are crucial for biological processes, such as differentiation [1], proliferation [2], and migration [3]. Changes in the cytoskeleton are often observed in certain diseases and can alter the response of cells to external stimuli and the environment [4]. This suggests that cell stiffness may serve as a potential indicator of pathological conditions. For instance, cancer cells are softer than normal cells, with membrane tension contributing to their malignancy [5], and this difference has been used to successfully separate diseased and healthy blood cells [6,7]. The close link between diseases and cell mechanics has driven ongoing research into the underlying mechanisms.

This link between cell mechanics and pathology is especially prominent in specific genetic disorders caused by defects in structural proteins. Emery-Dreifuss muscular dystrophy arises from dysfunctions in lamin A and emerin, proteins that play critical roles in nuclear signaling and structural maintenance [8,9]. Increased mechanical stress-induced apoptosis has been reported [10]. Similarly, a mutation in lamin A gene causes Hutchinson–Gilford progeria syndrome (HGPS), in which abnormal lamin A (progerin) accumulates within the nucleus. In healthy individuals, progerin levels also increase with age, contributing to nuclear stiffening [11] and cells exhibit increased mechanosensitivity [12]. Investigating these nuclear-related phenomena often requires applying significant strain and analyzing their overall mechanical properties, which can reflect changes within the nucleus. Consequently, understanding these conditions requires precise tools capable of measuring cellular mechanical properties.

Various devices and techniques have been developed to study the mechanical properties of cells [13,14]. One of the most traditional techniques is micropipette aspiration, which uses negative pressure to aspirate a part of a cell into a capillary, allowing evaluation of the stiffness of the cell under deformation [15,16]. However, precise measurements require careful positioning of the pipette, which may limit the types of samples that can be measured [17]. Another widely used technique is atomic force microscopy (AFM), in which a probe is pressed against a cell, and the resulting deformation is used to measure cell stiffness [18,19,20]. AFM is highly sensitive and provides high-resolution data; however, it requires cells to be fixed to a substrate, which makes it challenging to measure suspended cells [21]. Furthermore, they are unsuitable for measurements with large deformations. Optical tweezers use focused laser beams to manipulate cells without physical contact, thus enabling the measurement of mechanical properties by stretching the cells [22]. However, as the generated forces are on the piconewton scale, optical tweezers cannot apply sufficient force to induce substantial deformation [23]. Additionally, focused laser beams can potentially damage intracellular structures [24].

Among these, techniques that use two probes to manipulate cells have attracted considerable attention because of their versatility and ability to induce significant deformations [25,26]. Although these techniques can provide sufficient cell deformation, they are limited by their low degrees of freedom. This restricts the precise positioning and orientation of the end-effectors, making controlled and targeted measurements challenging. To overcome these limitations, we developed the ‘Microhand.’ The ‘Microhand’ is a high-resolution micromanipulator featuring a 3-DoF parallel link mechanism driven by piezoelectric actuators. This system operates two chopstick-like end-effectors, which are integrated with microforce sensors capable of nN order force measurement. This approach, which directly grasps samples with two end-effectors, enables the stiffness measurements of cells [27]. Furthermore, because the reaction force is measured directly by a microforce sensor, the direction of the force can be freely set, and the reaction force can be measured without observing the end-effector. However, when the end-effector is replaced, the sensor also needs to be replaced, which is a problem because of the time and effort required to perform the calibration each time. In addition, earlier studies primarily used needle-shaped end-effectors, which made it difficult to deform an entire cell.

Therefore, in this study, we first addressed the automation of microforce sensor calibration and subsequently investigated the importance of the end-effector shape in a microhand system. Specifically, we investigated how needle- and plate-shaped end-effectors affect the measured stiffness of human embryonic kidney (HEK293A) cells. Our results revealed that the larger contact area of the plate-shaped end-effectors enabled both substantial deformation and stable measurements. Using plate-shaped end-effectors, we evaluated the effects of various treatments such as cytoskeletal drugs on cell stiffness and characterized the viscoelastic properties of HEK293A cells. Furthermore, we employed a microhand system to assess the stiffness of HGPS model cells, revealing significant differences in stiffness distribution compared to healthy cells. Finally, we conducted cell indentation tests, which enabled separate evaluation of the mechanical properties of the cytoplasm at small indentation depths and the nucleus at large depths. These findings collectively demonstrate that our microhand system is a versatile and valuable platform for detailed evaluation of whole-cell mechanical properties.

## 2. Materials and Methods

### 2.1. System Configuration

**Figure 1:**
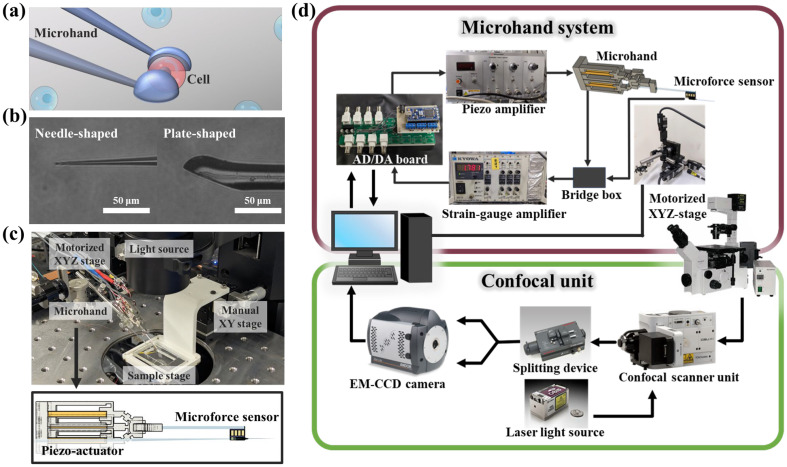
(**a**) Schematic illustration of the cell manipulation. (**b**) Side-view micrograph of the actual needle- and plate-shaped end-effector. (**c**) Perspective view of the experimental system setup and detailed CAD cross-section of the microhand. (**d**) System configuration diagram of the microhand.

We developed a two-fingered microhand system with plated-shaped end-effectors (Figure 1a,b). The system has two end-effectors: one side is equipped with an actuator and a microforce sensor, whereas the other side is passive, with no actuator or sensor (Figure 1c). The actuator features a parallel-link structure with three piezoelectric elements, and two strain sensors attached to each element for feedback control. Collectively, these components enable the precise 3-DoF manipulation. The sensor values of the system are acquired using an AD/DA board connected to a microcontroller, which also regulates the voltage supplied to the piezoelectric elements. The output voltage was amplified to the range of 0–150 V using a piezoelectric amplifier (HJPZ-0.15Px3, Matsusada Precision Inc., Shiga, Japan). The changes detected by each strain gauge and microforce sensor were amplified using a strain amplifier (MCD-16A, DPM-71A, Kyowa Electronic Instruments Co., Ltd., Tokyo, Japan). For imaging, a confocal scanner unit (CSU-X1, Yokogawa Electric Corporation, Tokyo, Japan) was attached to a microscope (IX-73, Olympus Corporation, Tokyo, Japan). Additionally, an image-splitting device (W-VIEW GEMINI, Hamamatsu Photonics K.K., Shizuoka, Japan) was used to acquire fluorescent and bright-field images simultaneously using an EM-CCD camera (Andor iXon Life 888, Oxford Instruments, Abingdon, UK). The microhand was mounted on a three-dimensional motorized stage (SGSP-25ACT-B0, Sigmakoki Co., Ltd., Tokyo, Japan) (Figure 1d).

### 2.2. Fabrication of End-Effectors and Microforce Sensors

**Figure 2:**
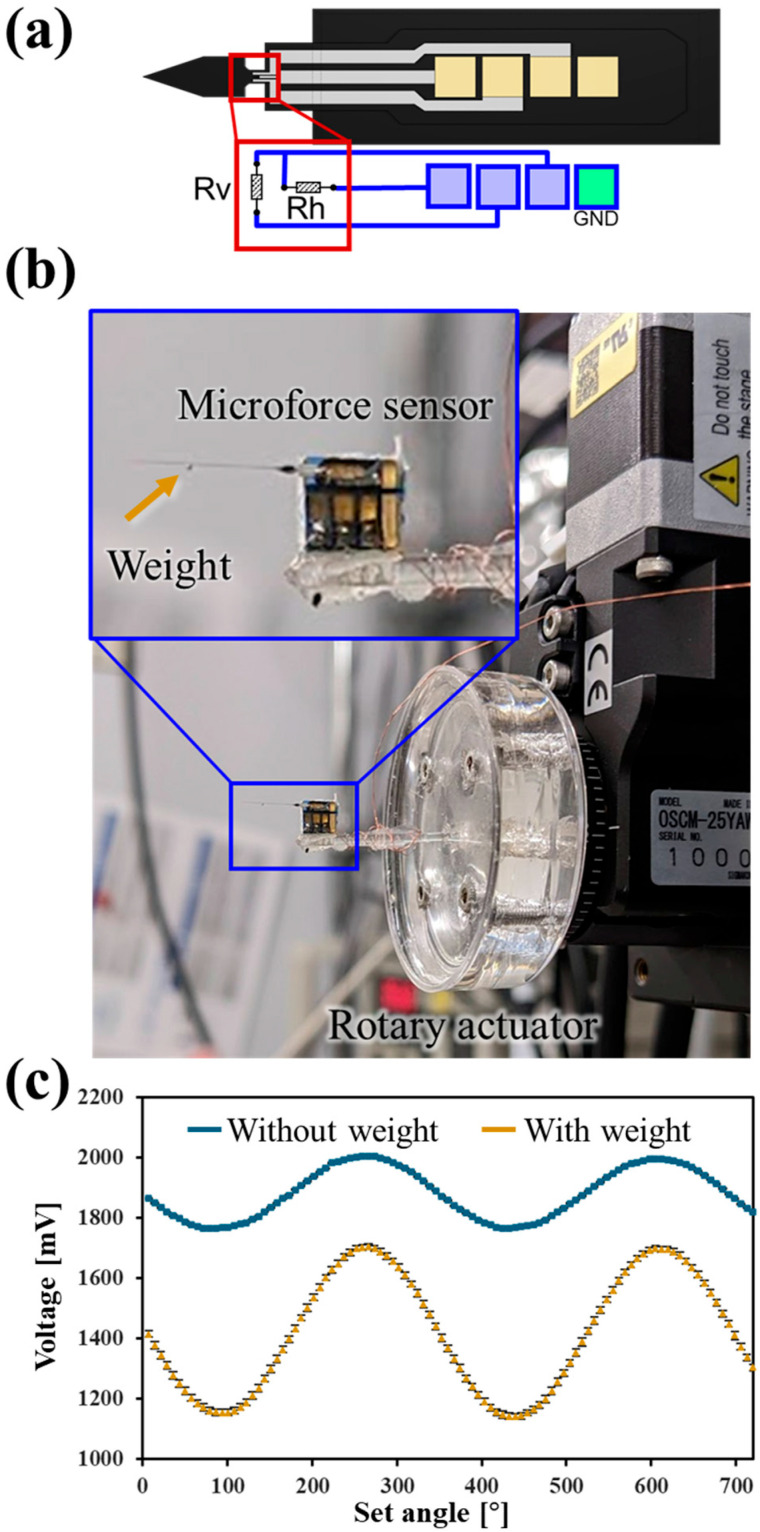
(**a**) Design of microforce sensor and strain gauges. (**b**) Experimental image of the assembled microforce sensor for calibration. (**c**) The A/D values data acquired during the calibration process. Error bars represent standard error (*n* = 50).

The fabrication of the end-effectors followed a specific process. A needle-shaped end-effector was created by pulling a heated glass rod (G-1000) with a puller (PC-100). To fabricate the spherical end-effector, the needle-shaped tip was heated using a microforge (MF2). The plate-shaped end-effector was produced by grinding the two faces of the spherical end-effector using a micropipette grinder (EG-402) (all equipment from Narishige Co., Ltd., Tokyo, Japan). Microforce sensors were fabricated by mounting semiconductor strain gauges onto the substrate and wiring. The tapered region of this strain gauge was designed to detect the applied forces with high sensitivity (Figure 2a). To secure the wiring, the area was fixed with an epoxy resin. A needle- or plate-shaped end-effector was affixed to the tip of the strain gauge, and the surface of the substrate was coated with an adhesive and left to dry for 24 h. Finally, a glass rod was attached as a handle to mount the sensor onto the manipulator.

### 2.3. Automated Calibration Method for Microforce Sensors

Each microforce sensor was calibrated before the experiments. A microforce sensor was attached to the Polydimethylsiloxane fixture, which was mounted on a motorized rotary actuator (OSCM-25YAW, Sigmakoki Co., Ltd., Tokyo, Japan). To apply a gravitational load, a chrome steel ball (3.27 × 10^−5^ g) was placed on the tip of the microforce sensor (Figure 2b). During the calibration procedure, the sensor was incrementally rotated through one full revolution in 50 steps corresponding to 7.2°/1 step. The output value of the sensor at each angular position was recorded (Figure 2c). Subsequently, the gravitational force component exerted by the steel ball along the sensitive axis of the sensor was calculated for each specific angle. The force-to-signal conversion coefficient was determined for the sensor by correlating the calculated applied forces with the corresponding recorded sensor output signals. A more detailed description is provided in a previous publication [28].

### 2.4. Materials

Cytochalasin D (CD), Trichostatin A (TSA), L-glutamine, Streptomycin were purchased from FUJIFILM Wako Pure Chemical Co., Ltd. (Osaka, Japan). CD was dissolved in 1 mL dichloromethane to a concentration of 1.97 mM and TSA was dissolved in 1 mL dimethyl sulfoxide (DMSO) to 1.00 mM. The LipofectamineTM 3000 reagent was purchased from Thermo Fisher Scientific Inc. (Waltham, MA, USA). Dulbecco’s modified Eagle’s medium (DMEM) was purchased from Shimadzu Diagnostics Co., Ltd. (Tokyo, Japan) and fetal bovine serum (FBS) was purchased from Peak Serum Inc. (Wellington, CO, USA). Thermoresponsive cell culture dishes, which changes its hydrophilicity/hydrophobicity at 32 °C were purchased from CellSeed Inc. (UpCell^®^, Tokyo, Japan). Nuclear Green LCS1 (ab138094) and Phalloidin-iFluor^TM^ 680 were purchased from Abcam (Cambridge, UK).

### 2.5. Cell Culture Condition

Human embryonic kidney (HEK293A), mouse myoblast (C2C12), human cervical cancer (HeLa), mouse embryonic fibroblast (10T1/2), and human bone marrow-derived stromal (UE7T-13) cells were cultured at 37 °C in DMEM supplemented with 10% FBS, 2 mM L-glutamine, and 100 U/mL streptomycin. The culture environment was maintained at 5% CO_2_ and 95% air, with high humidity.

### 2.6. Staining Process of Cells

Nuclear staining was performed using Nuclear Green LCS1. A stock solution (5 mM in DMSO) was diluted 1:1000 in phosphate-buffered saline (PBS), and 1 mL of this solution was added to the culture dish. The cells were incubated for 20 min. For F-actin staining, the cells were rinsed thrice with PBS, fixed in a paraformaldehyde solution, and incubated for 30 min. After fixation, the cells were incubated in HEPES buffer for 10 min and rinsed with PBS. F-actin was then stained for 90 min with Phalloidin-iFluor™ 680 (1 μL) in PBS (1 mL) containing 1% bovine serum albumin (BSA).

### 2.7. Overexpression of Lamin A and Progerin

Plasmids for overexpression of lamin A and progerin were named pEGFPC1-wt human lamin A (Lamin A OX) and pEGFPC1-human progerin (Progerin OX), respectively. Plasmids were constructed using PCR amplification. The amplified gene was cloned into the expression vector pEGFP-C1 (Clontech) and expressed as a protein that fused EGFP to the N-terminus. Transfection was performed using Lipofectamine^TM^ 3000 following the manufacturer’s instructions. For comparison of cell stiffness, normal HEK293A cells and Lamin A OX cells were used. Only cells exhibiting EGFP fluorescence were selected for stiffness measurement. The transfection efficiency was approximately 85% for Lamin A OX and 76% for Progerin OX.

### 2.8. Stiffness Measurement of Individual Cells

**Figure 3:**
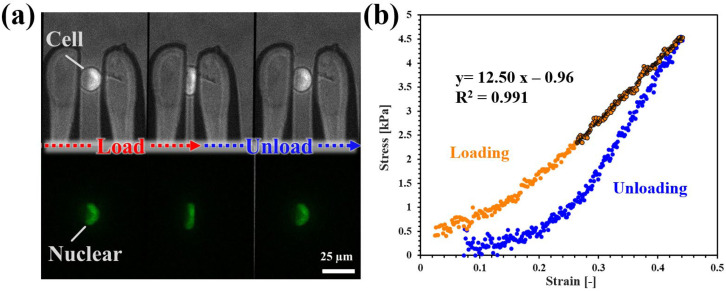
(**a**) Example of indentation testing of cells performed with the microhand. (**b**) Example of strain–stress curve during indentation test (orange: loading (going up), blue: unloading (going down) process). The elastic modulus was calculated from high-strain region.

Before conducting the experiments, the end-effectors were immersed in a BSA solution (1 *w*/*v* % in PBS) to minimize non-specific cell adhesion. Cells were cultured to subconfluence, trypsinized, and collected by centrifugation at 1000 rpm for 2 min. The cell pellet was gently resuspended in PBS. For treatment conditions, TSA was applied at concentrations of 250 or 500 nM in PBS for 3 h, while CD treatment was applied at 1 or 2 μM in PBS for 30 min. For stiffness measurements, each cell was individually grasped using a microhand system, and the reaction force was recorded along with a video of the compression process lasting one second (Figure 3a). The applied stress was calculated by analyzing the reaction force and determining the contact area from the captured images.

Contact length $D_{t}$ and cell width $L_{t}$ were measured from images (Figure S1). The contact area $S_{t}$ was approximated as a circle.

$$S_{t}=\frac{\pi {D_{t}}^{2}}{4}$$

Stress $\sigma_{t}$ was calculated from the measured force $F_{t}$ and contact area:

$$\sigma_{t}\;=\;\frac{F_{t}}{S_{t}}$$

Strain $\varepsilon_{t}$ was calculated from the deformation and the initial width $L_{0}$:

$$\varepsilon_{t}\;=\;\frac{L_{0}-L_{t}}{L_{0}}$$

To determine the elastic modulus, a linear regression analysis was performed on the data points in the high-strain region of strain–stress curve, and the plot exhibited clear linearity (Figure 3b).

### 2.9. Statistical Analysis

Data are presented as the mean ± standard deviation. Statistical analysis was conducted using Student’s *t*-test, and a *p*-value of less than 0.05 was considered significant.

## 3. Results and Discussion

### 3.1. Automation of Microforce Sensors Calibration

We previously reported a manual method for evaluating the microforce sensors [28]. In this method, sensors were mounted on a manual rotary manipulator, and their A/D output values were captured every 36° during rotation. However, this manual procedure had several limitations, including difficulties in precisely controlling the rotation angle and insufficient data density (10 points per rotation), both of which can compromise the calibration accuracy. To address these issues, we automated the calibration process by incorporating a motorized rotary stage. This enhancement allowed for finer angular sampling; specifically, we increased data acquisition from 10 to 50 angular positions per full rotation.

Because the sensing characteristics vary among individual sensors, we performed automated calibration to determine its specific conversion coefficient. Nine different sensors were examined, and the average resolution of the force sensor was determined to be 1.88 ± 0.28 nN (*n* = 9). The standard deviation of the measurements and the root mean squared error of the calibration fit were comparable with those obtained using the previous manual calibration method (Table 1, Figure S2). Furthermore, the automation reduced the maximum error between the predicted and measured values (Table 1). These results confirmed that the automated calibration method was effective and reliable. These findings indicate that microforce sensors calibrated using our automated method can detect forces with high precision.

### 3.2. Effect of End-Effector Shape on Cell Stiffness Measurement

Previously, we utilized needle-shaped end-effectors for the precise manipulation [29]. Although the needle-shaped end-effector is relatively easy to fabricate, it has limitations in achieving precise tip alignment and is less effective in inducing substantial cell deformation. To address these limitations, we redesigned the end-effector tip as a flat plate. HEK293A cells were used to demonstrate the differences in experimental outcomes. Subsequently, to validate the effectiveness of the plate-shaped end-effector, we applied significant deformations to the individual cells.

**Figure 4:**
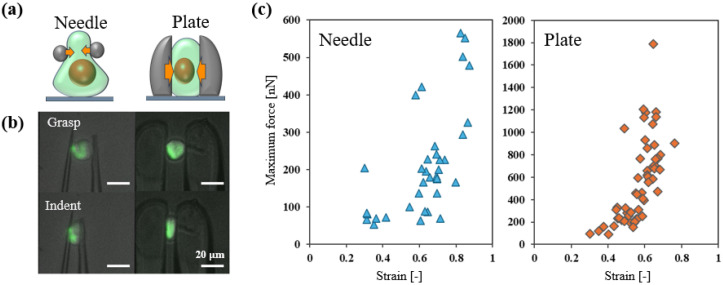
(**a**) The schematic diagram and (**b**) actual image of the cell compression using needle (**left**) and plate (**right**) end-effectors (green: nucleus). (**c**) The maximum force applied to the HEK293A cells by the needle-shaped (*n* = 30) and plate-shaped (*n* = 57) end-effectors were plotted.

The plate-shaped end-effectors were expected to impose significant deformation on the cell nuclei because of their larger contact area. This hypothesis was experimentally verified by fluorescence imaging, which clearly showed marked nuclear deformation upon compression by the plate-shaped end-effectors (Figure 4a,b). Analysis of the maximum force recorded during compression for each end-effector type revealed that the needle-shaped design yielded a broad distribution of forces. By contrast, the plate-shaped design exhibited a substantially narrower and more consistent force distribution (Figure 4c). Other studies have reported that cytoplasmic stiffness typically ranges from approximately 0.5 to 3.0 kPa [19], whereas the nucleus is estimated to be approximately ten times stiffer [20]. Therefore, the broad force distribution observed with needle-shaped end-effectors may reflect instability during whole-cell compression, particularly when involving the nucleus. To further investigate this, we observed the stained cell nuclei during compression (Figure 4b). These observations indicate that the plate-shaped end-effector successfully deformed the nucleus in 95.0% of cases (*n* = 20) compared to only 27.3% of cases (*n* = 11) for the needle-shaped end-effector. These findings suggest that plate-shaped end-effectors enable more stable indentation and, consequently, more reliable measurements of cellular mechanical properties. Compared with needle-shaped end-effectors, plate-shaped end-effectors are better suited for investigating the contributions from the cytoskeleton and related intracellular structures, especially under conditions of large deformation.

### 3.3. Mechanical Properties of Different Cell Types

**Figure 5:**
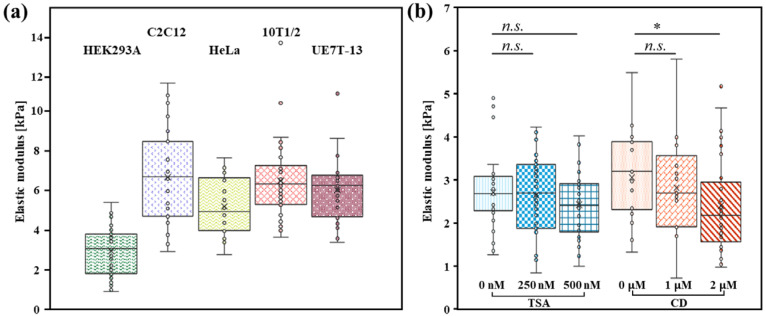
(**a**) Single cell stiffness differs dependent on the cell type. (**b**) Trychostatin A and Cytochalasin D decrease the cell stiffness. Statistical analysis was performed using Student’s *t*-test (* *p* < 0.05, *n.s.* = not significant). In the box plots, the ‘×’ symbol represents the mean, the center line indicates the median, the box limits represent the 25th and 75th percentiles, the whiskers extend 1.5 times the interquartile range from the 25th and 75th percentiles, and all individual data points are shown as dots.

In addition to the HEK293A cells, we measured the stiffness of C2C12, HeLa, 10T1/2, and UE7T-13 cells. The measured stiffness values were as follows: C2C12, 6.55 ± 2.46 kPa (*n* = 23); HEK293, 2.85 ± 1.15 kPa (*n* = 57); HeLa, 5.08 ± 1.47 kPa (*n* = 16); 10T1/2, 6.36 ± 1.96 kPa (*n* = 31); and UE7T-13, 5.90 ± 1.60 kPa (*n* = 31) (Figure 5a).

In previous studies using AFM with a conical tip, the reported stiffness for C2C12 cells was approximately 3.0 kPa, for HEK293 cells approximately 1.9 kPa [30], and HeLa cells 2.48 kPa [31]. These earlier findings indicate that C2C12 cells were the stiffest, whereas HEK293A cells were the softest, among the cell types measured in those studies. Despite the known sensitivity of absolute stiffness values to methodological differences (indenter shape, depth, equipment, and calculation methods), the relative stiffness ranking of the cell types was consistent between our results and previous studies. This suggests that our method reliably captures the qualitative differences in stiffness across different cell lines.

### 3.4. Comparison of HEK293A Cell Stiffness After Different Drug Treatments

To further understand the contribution of specific intracellular structures to overall cell stiffness, we investigated the role of F-actin. F-actin are recognized as major determinants of cellular mechanical properties. Therefore, we measured individual cell stiffness after treatment with Cytochalasin D (CD), an inhibitor of actin polymerization (Figure 5b). Disruption of F-actin reduces intracellular contractile forces and impairs cell spreading [32]. Correspondingly, the elastic moduli of the cells decreased in a concentration-dependent manner. The stiffness of untreated HEK293A cells was 3.05 ± 0.99 kPa (*n* = 26). Upon treatment with 1 μM CD, the stiffness decreased to 2.81 ± 1.16 kPa (*n* = 17), and at 2 μM CD, it further decreased to 2.39 ± 1.10 kPa (*n* = 29). These results confirmed that F-actin plays a significant role in maintaining cellular stiffness, even in suspended cells. Although a direct quantitative comparison is difficult due to different experimental conditions, our finding of a concentration-dependent stiffness reduction is qualitatively consistent with previous studies, which also reported a significant stiffness decrease using 5 μM CD [33].

We also examined the potential influence of chromatin organization on cell stiffness by treating the cells with Trichostatin A (TSA), a histone deacetylase inhibitor (Figure 5b). Chromatin, a complex of DNA and histone proteins, is a key structural component of the nucleus that interacts with the nuclear envelope and is crucial for maintenance of nuclear stability [34]. Histone deacetylation alters chromatin structure, leading to a more relaxed, deformable nuclear architecture [35,36]. Our experiments with TSA revealed a trend of decreasing cell stiffness with increasing inhibitor concentration, although this effect was less pronounced than that observed with CD (Control: 2.70 ± 0.86 kPa (*n* = 30); TSA 250 nM: 2.64 ± 0.92 kPa (*n* = 31); TSA 500 nM: 2.42 ± 0.76 kPa (*n* = 26)) (Figure 5b). As no statistically significant difference was observed in cell stiffness, it is considered that the contribution of chromatin to the overall cell stiffness is small compared to that of the F-actin.

The cellular stiffness measurements described in the previous sections were primarily conducted on cells harvested by trypsinization, a common enzymatic treatment that potentially alters cell surface properties [37,38,39]. To assess the effects of this procedure, we compared the stiffness of trypsinized HEK293A cells with that of non-trypsinized cells. The untreated cells were slightly stiffer than trypsinized cells; however, this difference was not statistically significant (Trypsinized: 2.84 ± 1.15 kPa (*n* = 57); non-trypsinized: 3.21 ± 1.09 kPa (*n* = 36)). This observation suggests that the extracellular matrix (ECM) components synthesized by cells in 2D culture, or the membrane proteins potentially degraded by trypsin, have minimal impact on the overall stiffness of suspended HEK293A cells, as measured by our system (Figure S3). However, the histogram of the non-trypsinized stiffness values reveals a slight shift in the overall cell stiffness toward higher values. These results indicate that the mechanical relationship between cells and ECM can be examined in detail using a microhand system by studying various cell types and experimental conditions.

### 3.5. Altered Mechanical Characteristics in HGPS Model Cells

HGPS is caused by a mutation in LMNA, which leads to defective nuclear lamina formation. This defect affects transcription processes and signal transduction, as a farnesylated prelamin, known as progerin, accumulates at the nuclear envelope without forming a proper lamina network [40,41]. This accumulation causes nuclear deformation and sclerosis, and reduces mechanical resistance [42].

**Figure 6:**
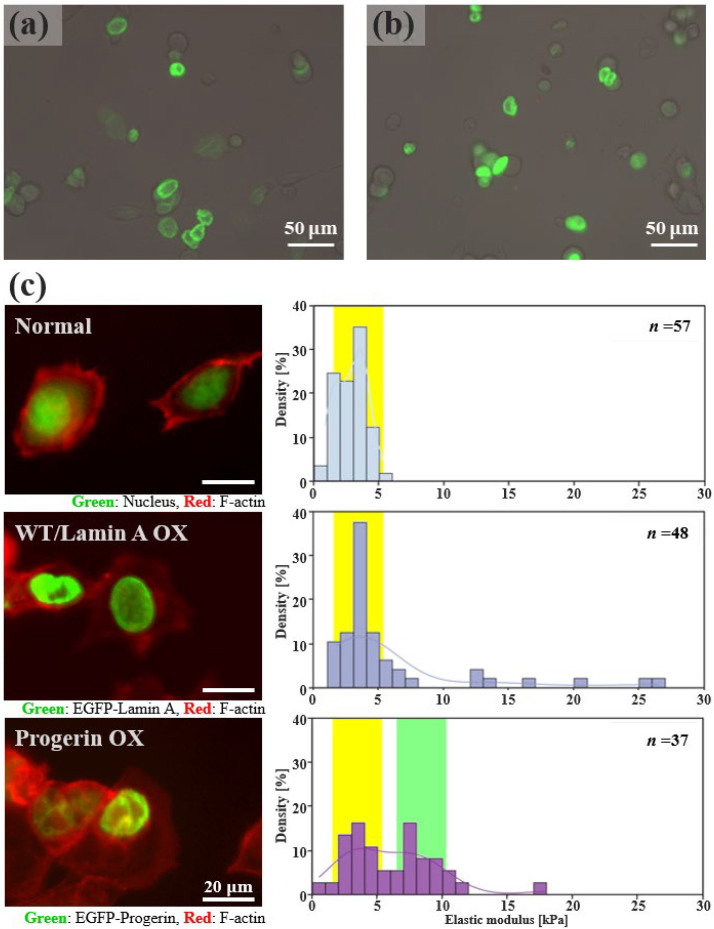
Overexpression of lamin A and progerin alters the morphology and stiffness of HEK293A cells. (**a**,**b**) Fluorescence images confirming the overexpression of EGFP-tagged wild-type lamin A and progerin in transfected cells. (**c**) **Left**: Comparison of cell morphology and actin cytoskeleton organization. Images show wild-type cells, Lamin A OX cells, and Progerin OX cells. **Right**: Histograms of the cellular Elastic modulus for each condition. The *y*-axis represents the density, corresponding to the fraction of cells per 1 kPa interval. The yellow region highlights the contribution of native lamin A, while the green region highlights that of progerin.

HEK293A cells transfected with an EGFPC1-human progerin plasmid were used as a pathological model of HGPS. Transfection was confirmed by the expression of green fluorescent protein (Figure 6a,b). There were no significant differences in nuclear shape between normal cells and Lamin A OX cells; however, Progerin OX cells exhibited wrinkles on the nuclear surface (Figure 6c: left). This is consistent with previous reports on HGPS pathological model cells [43]. Notably, the expression of progerin leads to a reduction in nuclear F-actin, which impairs the maintenance of nuclear shape [44]. Conversely, the proportion of cytoplasmic F-actin increases, altering the responsiveness of the nucleus mechanoresponse [45]. Therefore, we stained F-actin to investigate its organization. However, we did not observe any significant morphological changes in the F-actin cytoskeleton under different conditions (Figure S4).

Next, we examined the elastic moduli of the cells using a microhand system. Both Progerin OX and Lamin A OX cells exhibited a broader distribution of elastic moduli than normal cells (Figure 6c: right). The histograms revealed that all the samples had a prominent peak at 2–4 kPa, suggesting that this stiffness range primarily reflected the contribution of the endogenous cytoskeleton. Progerin OX also exhibited a peak at 6–8 kPa. The histogram of Lamin A OX showed a right-skewed distribution, suggesting a significant contribution of lamin A protein to whole-cell stiffness. We hypothesized that Lamin A OX increased cell stiffness by strengthening the existing wild-type lamin meshwork [46]. In contrast, progerin does not form lamina network. Instead, it accumulates on the nuclear envelope, resulting in an altered stiffness distribution. Our findings are consistent with those of previous micropipette aspiration studies reporting that the stiffness of the nucleus of a normal HeLa cell is ~4 kPa, whereas that of a progerin-expressing cell is ~9 kPa [47]. Therefore, we interpreted the lower peak (2–4 kPa) observed in Progerin OX cells as the contribution of native lamin A whereas the higher peak (6–8 kPa) reflected the progerin accumulation. This implies that, when progerin expression exceeds a certain threshold, its stiffening effect on the nuclear membrane dominates that on the endogenous network.

These results indicate that the microhand system with planar end-effectors is well suited for analyzing mechanical properties and detecting alterations linked to internal cellular structures. This system can detect subtle changes in mechanical properties that reflect these internal structural alterations, providing insights into their mechanobiological impact.

### 3.6. Viscoelastic Properties of HEK293A Cells at Different Indentation Speed

**Figure 7:**
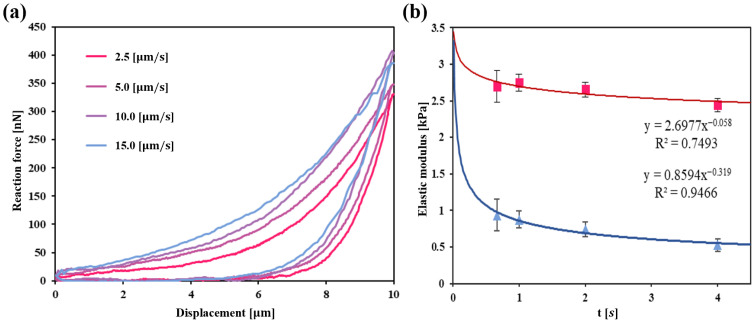
(**a**) Force–displacement curve at different indentation speed. (**b**) Comparison of elastic modulus fitted with the power-law model. The red data shows high-compression conditions (nucleus) and the blue data shows low-compression. Error bars represent standard error (*n* ≥ 16).

To investigate the viscoelastic nature of HEK293A cells, we evaluated the mechanical response while varying the indentation speed from 2.5 to 15.0 µm/s (Figure 7a). We then compared viscoelastic behavior under two distinct conditions: low compression (predominantly probing the cytoplasm) and high compression (reaching and indenting the nucleus). Viscoelastic properties were analyzed using the following power-law rheology model [48]:

(1) $$E\left(t\right)\;=\;E_{0}{\left(\frac{t}{t_{ref}}\right)}^{-\beta }$$

where *E*_(*t*)_ is the time-dependent elastic modulus, *E*_0_ is the initial elastic modulus, *t_ref_* is the reference time (set to 1 s), *t* is the indentation time, and *β* is the power-law exponent. A *β* value of 0 signifies a purely elastic solid with an Elastic modulus of *E*_0_, while a *β* value of 1 corresponds to a Newtonian viscous liquid. By fitting this model to the experimental data, we extracted the parameters *E_0_* and *β* for each condition.

Our analysis, using the power law model, showed that HEK293A cells exhibited a higher constant elastic modulus at greater indentation depths (nuclei) than at shallower depths (cytoplasm). Furthermore, the power-law exponent *β* indicated that the nucleus displayed more solid-like (elastic) properties, whereas the cytoplasm demonstrated more fluid-like (viscous) characteristics (Figure 7b). This trend suggests distinct mechanical behaviors in the cytoplasm and nucleus, with each compartment contributing uniquely to the overall cellular mechanical response. These findings demonstrate that the microhand system developed in this study can effectively distinguish between the mechanical properties of the nucleus and cytoplasm.

## 4. Conclusions

In this study, we developed a microhand system equipped with two end-effectors designed for the precise manipulation and mechanical characterization of single cells. Through a comparative analysis, we demonstrated that plate-shaped end-effectors provide superior performance in controllably deforming cells and accurately measuring their stiffness, thereby enabling effective mechanical interrogation of the nucleus. This finding was experimentally validated using multiple cell types. These findings underscore the importance of the end-effector shape in single-cell mechanical studies, as the plate-shaped design facilitates a larger and more uniform contact area and controlled compression, yielding more reliable and reproducible stiffness data. Additionally, by applying treatments with Cytochalasin D and Trichostatin A investigated the contribution of key cytoskeletal components and chromatin structures to cell stiffness. Our system successfully detected the expected alterations induced by these reagents, which is consistent with the established literature. Furthermore, our investigation into the effects of progerin overexpression in HEK293A cells provides insights into how nuclear stiffening associated with HGPS can be quantitatively assessed, offering a new perspective on the mechanical underpinnings. Finally, we assessed cellular viscoelastic properties and revealed distinct mechanical signatures in the cytoplasm and nucleus. This observation highlights the complex and heterogeneous mechanical landscape within the cells, which can be further explored using our microhand system.

Despite these advancements, several challenges still need to be overcome. First, the throughput of cell stiffness measurements requires further improvement. The current system is also incapable of performing dynamic mechanical tests such as applying periodic stimulation or conducting indentation at high strain rates. Finally, a deeper understanding of cellular mechanobiology will necessitate the integration of our system with multicolor fluorescence imaging to track the dynamics of key molecules. In conclusion, the microhand system and approaches presented in this study offer a versatile and robust platform for precise mechanical measurements of single cells. This system can be extended to investigate a wide array of cell types, diverse mechanical phenomena, and cellular responses under various physiological and pathological conditions. Future studies employing this system are needed to further elucidate the pathogenesis of conditions such as HGPS and to unravel the complex intracellular mechanical phenomena. Addressing these limitations will further enhance the applicability of this promising technology.

## Figures and Tables

**Table 1:** Root mean squared error (RMSE) and maximum error of microforce sensor.

	Manual Method [28]	Automated Method
RMSE [nN]	3.87	5.38
Maximum error [nN]	25.4	10.7

## Data Availability

The data that support the findings of this study are available from the corresponding author, upon reasonable request.

## Supplementary Materials

The following supporting information can be downloaded at: https://www.mdpi.com/article/10.3390/mi16111272/s1, Figure S1: Schematic diagram illustrating the parameters used for stress and strain calculation, showing the transition from the initial state (left) to the deformed state (right). The initial state (*t* = 0) defines the initial cell width (L_0_) and initial contact length (D_0_), while the deformed state shows the deformed width (L_t_) and the contact length (D_t_) with the end-effector.; Figure S2: Scatter plot of predicted versus measured values of manual method (a) and automated method (b) for evaluation of calibrated sensors. Data alignment along the y = x line indicates the accuracy of the prediction.; Figure S3: Comparison of cell stiffness under trypsinized (*n* = 57) and non-trypsinized (*n* = 36) conditions. (a) Histogram showing a similar distribution of stiffness values in both conditions. (b) Box plot indicates that cells under the non-trypsinized condition were slightly stiffer than those under the trypsinized condition.; Figure S4: Fluorescence microscopy images of transfected HEK293A cells. (a) Control HEK293A cells. (b,c) Cells overexpressing EGFP-tagged lamin A and progerin, respectively. Red fluorescence indicates F-actin, and green fluorescence indicates the nucleus. Scale bar = 50 µm.
