# Supplementary material for: Microhand Platform Equipped with Plate-Shaped End-Effectors Enables Precise Probing of Intracellular Structure Contribution to Whole-Cell Mechanical Properties
Source: Micromachines (Basel). 2025 Nov 12;16(11):1272. doi: 10.3390/mi16111272 (PMC12654860; doi:10.3390/mi16111272)
Supplement: Supplementary file 1 [file micromachines-16-01272-s001.zip › micromachines-3955979-supplementary.docx]

*Supplementary Materials*

Microhand Platform Equipped with Plate-Shaped End-Effectors Enables Precise Probing of Intracellular Structure
Contribution to Whole-Cell Mechanical Properties

Masahiro Kawakami ^1^, Masaru Kojima ^1,^*, Toshihiko Ogura ^2^, Atsushi Kubo ^3^, Tatsuo Arai ^4^ and Shinji Sakai ^1^

^1^ Department of Materials Engineering Science, Division of Chemical Engineering, Graduate School of
Engineering Science, The University of Osaka, 1-3 Machikaneyama-cho, Toyonaka 560-8531, Osaka, Japan; kawamasa@cheng.es.osaka-u.ac.jp (M.K.); sakai@cheng.es.osaka-u.ac.jp (S.S.)

^2^ Center for Cancer Immunotherapy and Immunobiology, Kyoto University, Yoshida-Konoe-cho,
Sakyo-ku 606-8501, Kyoto, Japan; ogura.toshihiko.4n@kyoto-u.ac.jp

^3^ Graduate School of Pharmaceutical Sciences, The University of Osaka, 1-6 Yamadaoka, Suita 565-0871,
Osaka, Japan; kubo.atsushi.a3x@osaka-u.ac.jp

^4^ The Center for Neuroscience and Biomedical Engineering, The University of Electro-Communications,
1-5-1, Chofugaoka, Chofu 182-8585, Tokyo, Japan; tarai118@jcom.zaq.ne.jp

***** Correspondence: kojima@cheng.es.osaka-u.ac.jp


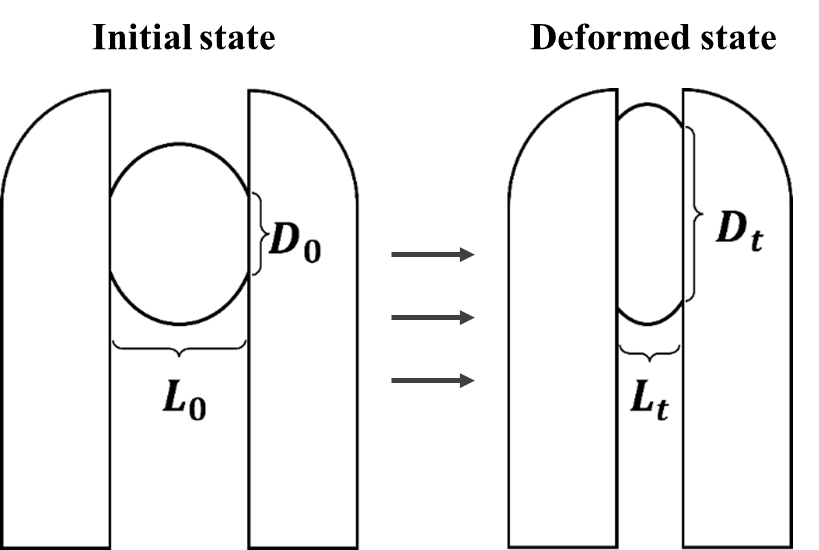


**Figure S1.** Schematic diagram illustrating the parameters used for stress and strain calculation, showing the transition from the initial state (**left**) to the deformed state (**right**). The initial state (t = 0) defines the initial cell width (*L*_0_) and initial contact length (*D*_0_), while the deformed state shows the deformed width (*L_t_*) and the contact length (*D_t_*) with the end-effector.


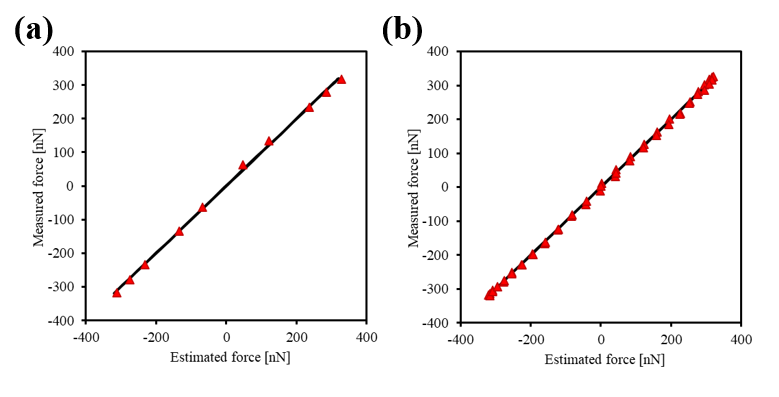


**Figure** **S2.** Scatter plot of predicted versus measured values of manual method (**a**) and automated method (**b**) for evaluation of calibrated sensors. Data alignment along the y = x line indicates the accuracy of the prediction.


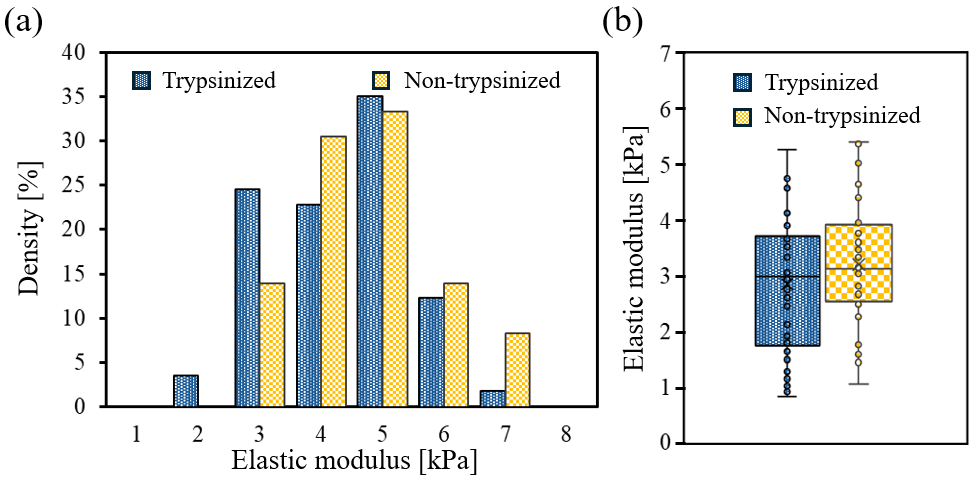


**Figure S3.** Comparison of cell stiffness under trypsinized (*n* = 57) and non-trypsinized (*n* = 36) conditions. (**a**) Histogram showing a similar distribution of stiffness values in both conditions. (**b**) Box plot indicates that cells under the non-trypsinized condition were slightly stiffer than those under the trypsinized condition.


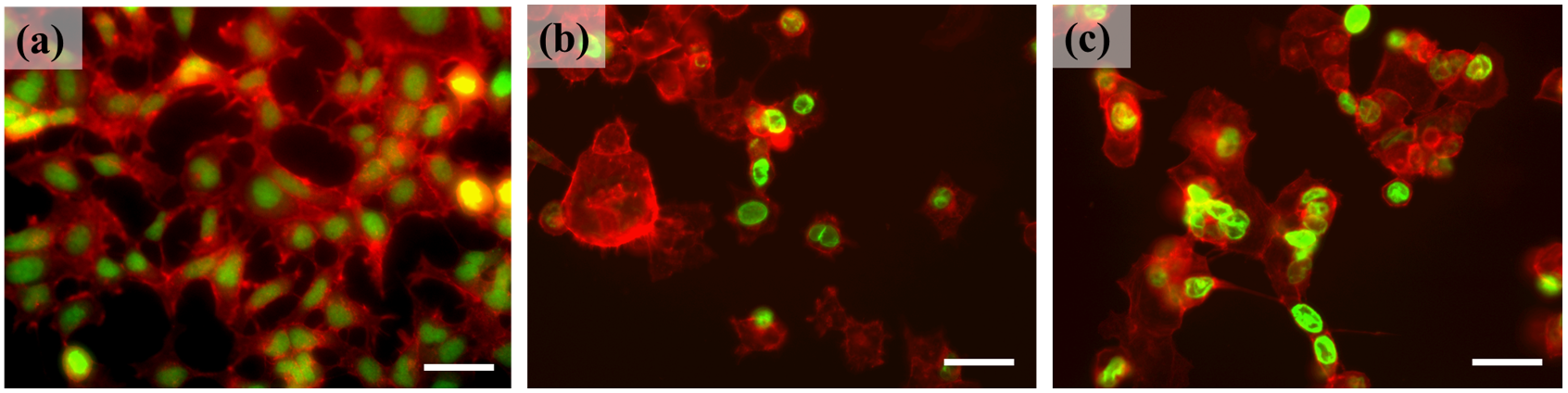


**Figure S4.** Fluorescence microscopy images of transfected HEK293A cells. (**a**) Control HEK293A cells. (**b**,**c**) Cells overexpressing EGFP-tagged lamin A and progerin, respectively. Red fluorescence indicates F-actin, and green fluorescence indicates the nucleus. Scale bar = 50 µm.
